# Understanding the psychological aspects of the radicalisation process: a sociocognitive approach

**DOI:** 10.1080/20961790.2020.1869883

**Published:** 2021-03-17

**Authors:** Serge Garcet

**Affiliations:** Interpersonal Criminology and Victimology, Department of Criminology, Faculty of Law, University of Liège, Liège, Belgium

**Keywords:** Forensic sciences, forensic psychology, radicalisation, sociocognitive paradigm, personality, social cognition, moral disengagement, relative deprivation

## Abstract

Understanding the psychological dimensions underlying the radicalisation process is a considerable challenge in the context of the legal judicial treatment of perpetrators of acts of radical violence. This clinical and qualitative study is based on an analysis of legal expert interviews of people at different stages of the radicalisation process. It highlights common psychological characteristics in personality, mechanisms of moral neutralisation and sociocognitive and interpretive treatment during radicalisation. These observations suggest the applicability of a model of cognitive–emotional transformation of self and meaning-building in radical violent engagement.

## Introduction

Research on violent radicalisation has developed from separate fields that have inevitably emphasised different radicalisation factors and highlighted different topics. A systematic scoping review of 148 studies between 2001 and 2015 on the transformation of radicalisation into violent extremism [1] identified three main factors in the scientific literature on radicalisation: push, pull and personal factors. Push factors refer to structural roots that lead to terrorism, such as loss of legitimacy, geopolitical factors, state repression, relative deprivation, inequality, intergroup contact, injustice and violence. Pull factors, which are considered the main radicalisation factors, are aspects that appeal to extreme groups and lifestyles, such as group belonging, group mechanisms and incentives. The authors of the review add to this category what they call “cognitive factors”, such as consumption of propaganda, cultural congruence, perceived efficacy, group morality and adventure seeking. Personal factors include individual psychological characteristics that make the person more vulnerable (mental health conditions, depression, trauma), personality traits and individual demographic characteristics. If this three-factor structure corresponds fairly well to the segmentation of academic research fields, it corresponds very poorly to the ways in which a person constructs their relationship to the world. Therefore, a new integrative perspective, based on sociocognitive understanding, is needed to describe the interactions between these different factors. Indeed, the dominant approach in radicalisation research has long reduced personal factors to a possible psychopathological or sociodemographic fragility and defined the individual as a constitutive element of a social structure, who is passively influenced by push and pull factors. Most importantly, the dominant approach fails to take into account the role of the interpretive system. This system is based on the abstraction and symbolisation capacities generated by mental representations of the self and the world; these permit various cognitive operations of selection, categorisation and attribution and allow an individual to direct his/her actions toward personal goals that he/she values.

It has been established that psychopathological and psychiatric approaches specific to personal factors, as described above, have failed to identify a profile connected to mental illness or psychopathological structures [2–8]. Nor have psychoanalytical approaches [9, 10] demonstrated heuristic value. As Taylor and Quayle [11] point out: “*the active terrorist is not discernibly different in psychological terms from the non-terrorist; in psychological terms, there are no special qualities that characterize the terrorist”*. The same is true of radicalised people. This difficulty in identifying, at the psychological level, the origin of the radical process that leads to violent acts ultimately led to an epistemological break; the question of “why” in relation to the act having been set aside for “how”, consistent with an interactional and processual rationale [12].

The sociocognitive framework of personality has been broadly validated. The use of this framework permits a return to the psychological aspects of radicalisation through mental operations (processes) and sociocognitive content (e.g. representations, schemas, distortions) that constitute the person’s interpretive system [13]. Indeed, “*individual radicalisation takes place during the changing phase in which a combination of reflection, knowledge acquisition and identity reassessment occurs. Changes in behaviour (including violent behaviour) is one product of the outcome phase and is a reflection of the solidification and empowerment of the individual’s new meaning perspective, belief system, and identity*” [14]. Taking a sociocognitive approach to radicalisation involves questioning the effect of the person’s information processing on his/her own cognitions and environment during the radicalisation process. In his article *Understanding the Terrorist Mind-Set* [15], Borum states that “*All people operate on their own internal ‘map’ of reality, not reality itself. This is a mental-behavioral phenomenon that psychologists refer to as ‘social cognition’. If people understand their opponents’ ‘maps’, it becomes easier to understand and to anticipate their action.*” Each individual has a capacity for self-determination that allows them to intentionally influence their environment by adapting their behaviour, emotions, goals and values to the situations encountered [16]. This is achieved by arranging the environmental conditions so that they are most conducive to producing the indicated behaviour and by creating cognitive aids and self-reinforcing aids to support this behaviour [17]. This capacity is developed through the person’s idiosyncratic interpretive system. The resulting action defines the individual’s behavioural signature and personality [18].

This subjective interpretive system also forms the basis of self-reflection, which allows individuals to anticipate their actions and assess the personal impact of their experiences and their effect on the situations in which they directly or indirectly participate. These capacities for self-organisation, self-reflection and self-regulation that underpin self-efficacy beliefs are at the heart of the capacity for self-determination that Bandura called human agency [19]. Of course, agency is exercised within the limits and constraints imposed by the reciprocal interactions between individual, behavioural and environmental factors. This reading of social cognition therefore goes further than the identification of vulnerability or risk factors (as in the push–pull factors model), as it postulates that there is a dynamic and permanent interaction between internal and external factors. These factors influence and change reciprocally in a contingent, interdependent and variable manner, over time and in different situations.

The cognitive-affective personality system (CAPS) proposed by Mischel and Shoda [20, 21] represents this interpretive system as a set of five types of cognitive-affective interconnected units. These units constitute mental representations (in the sense mentioned above) and schemas; that is, organising principles that explain lived experiences and guarantee a stable perception of oneself and the world. Cognitive-affective units are made up of (1) categorisations and definitions of oneself, of others and of the world. These are based on personal experience and aspects of the environment (e.g. family, culture, peers, society). These different “data banks” are organised according to different implicit theories of personality, stereotypes or prototypical readings. These categories are subject to various interpretive, inference and attribution biases, including hostile world schemas and attribution of hostility toward the exogenous group [22, 23]. They are also structured according to the positive or negative valence of the (2) emotions associated with them. The CAPS also produces (3) expectations and beliefs about social relationships and a personal sense of self-efficacy. The principles that govern moral judgment are part of these mental representations. Goals and values (4) are developed around projections of oneself in the future and goals or life projects associated not only with expected and desired positive consequences, but also with negative consequences and the fears that accompany these representations and affect the feeling of self-efficacy. Finally, there are (5) cognitive processes of self-organisation, self-reflection and self-regulation. In this sense, the cognitive mechanisms of moral neutralisation [24–26], which allow the person to avoid cognitive dissonance between performed acts and representations or beliefs by modifying the self-perception of individual responsibility, can be seen as stemming from the action of these different sociocognitive units. Bandura [27–29] identifies eight mechanisms of moral neutralisation; these are divided into three groups that focus on the perception of the transgressive behaviour, on the link between an action and its effect and finally on the person who became the object of the transgressive act.

Like any other type of behaviour, radicalisation can be considered a process in which the reciprocal determinism that guides the relationships between the person, their behaviour and their environment permanently modifies the person’s whole interpretive system (the CAPS). Radicalisation is therefore identified by a progressive and continuous transformation of representations, goals, beliefs and sources of reinforcement. Using this sociocognitive approach, it is therefore possible to go beyond the mechanistic model of radicalisation, which comprises a juxtaposition of push, pull and personal factors, and to propose a model that integrates push and pull factors within the person’s interpretive system and elucidates their possible influence in the process of radicalisation.

## Materials and method

### Population

The difficulty of accessing the relevant study population limits research on radicalisation. Another inherent problem with this type of research is selecting inclusion criteria appropriate to the radicalisation process, which can be defined as “*a process through which people become increasingly motivated to use violent means against members of an out-group or symbolic targets to achieve behavioural change and political goals*” [30]. Although it may be easy to identify people who have in one way or another participated in terrorist-like activities or have been identified by the legal and penitentiary systems as radicalised, it is more difficult to recognise where the radicalisation process begins and from what moment a person should be considered part of this process. However, in this study, we assumed that there is no gap between a commonplace thought and a radical thought, and that radicalisation is a continuum characterised by a progressive exacerbation of representations and values (cognitive radicalisation), which can be associated with different types of behaviour (behavioural radicalisation). Therefore, we thought it is important to analyse the development of the cognitive processes that underlie the radicalisation process as soon as they appear in thought or discourse. These cognitive processes may indicate an intellectual attraction to the themes advocated by the radical cause as soon as an orientation to the cause appears. We included subjects who only expressed cognitive adherence to the arguments of the Islamic State, which is consistent with the literature on radicalisation processes. Indeed, to explain the radicalisation process, Borum [31, 32] and Moghaddam [33] refer to such subjects in terms of their relative deprivation, and explain the first stage of the radicalisation process by a critical positioning of the subject within his/her environment. Wiktorowicz [34] suggests that a prior cognitive openness of the person is needed in addition to several other key components of mobilisation; namely, the socialisation process, non-coercive radical discourse and a search for meaning through religion. This cognitive openness may be triggered by a personal crisis or may be associated with previous social experiences of discrimination or victimisation. In the same way, McCauley and Moskalenko [35] consider a first radicalisation level that comprises sympathisers who adhere to the radical cause without endorsing violence. The integrative model of Ponsaer et al. [36] and the “sensitivity” phase in the model of Doosje et al. [30] feature the same integration of subjects at the threshold of the radicalisation process. We therefore included in the study individuals who adhered to a radical cause and exemplified cognitive radicalisation but not the subsequent expression of behavioural extremism.

This research used a qualitative and inductive clinical approach to study 27 people characterised by their personal level of involvement in the radicalisation process. The study used pre-trial judicial psychiatric and psychological reports and is ongoing. All the study subjects were Belgian but of Maghreb origin and aged from 17 to 31 years (mean: 24.6 years). All subjects defined themselves as Muslims but were unable to clearly state which religious current they followed; for instance, all but four could not say precisely if they were Salafist or not. The religious knowledge of most of the subjects was very poor and stereotyped. They all referred to the “Islamic State” as a single entity, without identifying with any specific faction or group commitment. The subjects had participated in military operations (returnees) or in acts labelled as terrorist (five subjects). Some subjects had been screened by the prison administration and incarcerated for reasons linked to radical issues (nine subjects). Six subjects had been identified while in custody as having radical attitudes, although they had not been prosecuted for infringements linked to radicalism/terrorism. Finally, the sample included seven subjects who had not been identified as radicalised by the legal and prison systems but whose affirmation of religious identity and statements indicated their support of the radical Islamic cause when interviewed by an expert. The sample therefore provided a collection of testimonials from individuals at different stages of the radicalisation process, prior to the full involvement of the legal and prison systems. Our objective was to propose a model of the cognitive processes underlying the different approaches cited above, so it was important to obtain a sample relevant to those approaches. Finally, from a medical–forensic perspective, it is important to recognise that sensitivity to radical ideas is a vector of radicalisation in imprisoned people. We felt that it was scientifically relevant to take into account this intellectual proximity to better understand the cognitive processes that gradually neutralise moral inhibitions and reinforce radicalisation even in the first stage.

### Methods

All the expert interviews were conducted according to a similar protocol that included a clinical component and psychometric and projective tests. The same person interviewed subjects and administered the psychological assessment in prisons in the south of Belgium (18 subjects) and in a private professional office (nine subjects). Subjects were interviewed in the context of a unique conference lasting for an average of 2 h. The interviews were not recorded, but written notes were taken.

The clinical approach we used comprised an anamnestic examination in the form of a study of the life course. It addressed family, educational, professional, relational and emotional spheres. The information in the person’s file was also mentioned regardless of whether these related to the issue of radicalisation. The approach also comprised an examination of the person’s medical and psychological history. In addition to this anamnestic examination, the interview included a discussion on radicalisation to situate the subject’s personal trajectory and representations at the time of the interview. The mechanisms of moral disengagement used by the subject in relation to radicalisation were also analysed.

The psychometric approach comprised the administration of several scales and inventories including the matrices of the Wechsler Adult Intelligence Scale (WAIS)-III for adults [37]; the Mini International Neuropsychiatric Interview [38]; the Symptom Checklist 90 (SCL-R90) [39]; the International Personality Disorder Examination for the Diagnostic and Statistical Manual of Mental Disorders, 5th Edition (DSM-V) [40], and the 10th revision of the International Classification of Diseases (ICD-10) [41]; the Young Schema Questionnaire (YSQ-L2/SQ2) [42]; Rorschach’s psychodiagnostic projective tests [43] and Murray’s Thematic Apperception Test [44]. Other scales were administered according to the requirements of the expert interviewers.

Two types of information were therefore collected. The first type is descriptive: it corresponds to the results of the various tests and inventories and describes the population encountered. The second type of information, the subject of this article, is based on an analysis of the subjects’ radical discourse and the description that they provided of their life path.

## Results

### Characteristics of the interviewed subjects

Subjects were young men ranging in age from the end of adolescence to approximately 30 years. During their adolescence, 20 subjects had frequently or infrequently presented with behaviour disorders as defined by the DSM-V/ICD-10 [40, 41], the intensity of which ranged from mild to severe, with limited prosocial emotions. Personality disorders were observed in 23 subjects, similar to the general prison population. The other subjects regularly presented features of specific personality types but did not demonstrate a long-term pervasive personality disorder. In terms of DSM-V/ICD-10 classification, the personality types most frequently observed were narcissistic (16 subjects), antisocial/dissocial (19 subjects), and borderline/emotionally labile, impulsive and borderline (11 subjects). Only three subjects had psychiatric disorders in the form of schizoid personality, schizophrenia and delusional disorders. Most subjects had double diagnoses of personality disorders. According to Young’s classification [42], schemas that refer to preferential patterns of information processing, the strictness that conditions dysfunction, are most often characterised by “impaired limits”; that is, these individuals *have not developed adequate internal limits in regard to reciprocity or self-discipline*. Such individuals have an egocentric and weakly empathetic character and difficulty implementing frustration management.

We also used Bandura’s model of cognitive mechanisms of justification and moral neutralisation. According to Bandura, moral disengagement is translated into various mechanisms that are classified into three groups depending on whether they deal with the perception of aggressive behaviour, the link between the act and its effect or the effect on the person or group that is the subject of the transgressive act. The mechanisms observed related mostly to perceptions of violent behaviour and the victims of the act. “Moral justification” by reference to a social need (defence of the Muslim community) and values (in the form of a reference to God and His Law) makes any actions taken socially acceptable and personally valued. The “advantageous comparison” mitigates the behaviour by comparing it to supposed immoral attitudes of the Western toward Muslims. Regarding the third group, which relates to perception of the victims, mechanisms of dehumanisation were observed. They included divesting the victims of human qualities so that they are seen as objects. “Attribution of blame” or criticism of the victim was also often used. This mechanism allows individuals to divest themselves of responsibility by defining themselves personally or through the group as belonging to a group of true victim(s) of the act committed. Finally, “euphemistic labelling”, which consists of sanitising the rhetoric, was also often expressed in statements of the persons interviewed.

### Speech analysis

Data analysis was carried out according to the principles of grounded theory. After coding the data from each subject, data were categorised according to the following criteria: victim positioning, the presence and recurrence of moral disengagement processes, positioning in relation to violence and its legitimacy, positioning in relation to the environment and what gradually constitutes the exogenous group, the affirmation and expression of radical representations and violent behaviour and the empowerment of discourse in relation to societal values. These criteria were then linked to integrate the data into clusters. The analysis revealed different key moments during the radicalisation process. We grouped the observations into five stages according to the evolution of the psychological processes used in the subjects’ conduct and their interaction with their environment. These clusters or stages formed the basis of our model of the radicalisation process (Table 1).

**Table 1.** Interactions among the environment, behaviours and cognitive processes observed during the stages of fascination, cause orientation, identity adherence, identity activism, and terrorist involvement.

Stages	Interactions among the environment, behaviour and cognitive processes
Fascination	**Subjective analysis of specific attributes of the radical cause** depending on experience, culture, personal life events. **Positive value attribution**, to the radical statements perceived as exterior. The attribution is based mostly on individual considerations such as an attachment to a relative, to a peer group, the desire to be recognized, the need of excitement and adventures. **Justification of existential attitude** by means of external attributions (injustice in the world) which allowed (1) to avoid cognitive discrepancy and discomfort resulting from a gap between aspirations and capacities to realize them (2) to transform advantageously the sense and to use negative emotions and feelings (anger, frustration) to express a right and legitimate revolt. **Reformulation of moral basis** in order to trivialize the use of group violence which arouses fascination. **Elaboration of positive self-reinforcing representations** that support the adequacy between the legitimacy of radical discourse and a violent action.
Cause orientation	**Active steps in quest for information**. The proximity with the desired cause induced by cognitive restructuration serves as a motive and as a reinforcement to continuation and amplifies the behaviour that arises from it. **Appearance of signs related to identity**. They define first of all at this stage a claim of adherence in the context of social environment. Later they tend to disappear taking into account their contradictions in relation to activism goals. **Orientation of behaviour and establishment of environmental conditions** necessary to get closer to positively marked radical topics and to assimilate them (self-conferring of status, familiarity with discourse contents…). **Repetition of behaviour.** The person behaves in the same way because he/she thinks that his/her actions will lead to the same results and consequences. The positive value of reinforcements which are obtained as a result of his/her actions condition the probability of the repetition of this behaviour.
Identity adherence	**Continuation of cognitive restructuration** which started at previous stages, namely as an increasing polarization. **Distancing from traditional referents**, from the family, from the community of origin, which are perceived as external. **Sounding board of peer group**. The person is not only reinforced by his/her own representations of himself or by the impact that he/she thinks to have on the environment, but also by the positive opinion of exterior observers whose adherence to the cause amplifies the value of reinforcement. **Internalization of discourse**. Exceeding the attitude of the fascination phase in which radical topics remained exterior to the person in order to assimilate radical topics and gradually merge with the identity references towards the logic of collective frameworking. **Reinforcement of cognitive mechanisms of moral neutralization by the group**.
Identity activism	**Identity related activism which is increasingly outrunning social and democratic norms (peri-democratic activism)** low and middle intensity actions, provocations, threats, support of radical actions of the group (concealment, spotting) and eventually violent attacks (for instance in response to expected reinforcements of the group leaders). **Neutralization of ostentatious identity signs**. **Moving to social exclusion and marginalization** often related to delinquency and/or secrecy. These individuals could become contact persons supporting logistics in case of necessity (weapons, hideouts, documents, money…). **Relatively important latency**. A noticeable age gap of several years between teenagers who get involved in radicalisation process and older individuals taking part in terrorist activities. **During the years of conflict voluntary departures to Syria and Iraq were taking place at this stage** even if beginning from the fascination stage spontaneous departures can be observed following the logic of attachment to the individuals who are already involved to a greater extent.
Terrorist involvement	**Multiple forms of involvement and modes of actions**: terrorism, armed actions in war zones. Sociocognitive **evasion** of **inhibitory mechanisms** set up by stays abroad. **Individuals’ difficulties to go back** since it would imply to call into question all the cognitions elaborated during the radicalisation process in the context of extreme existential crisis (cognitive discordance) as at this stage we observe an undifferentiation between personal and collective objectives.

**Figure 1.**
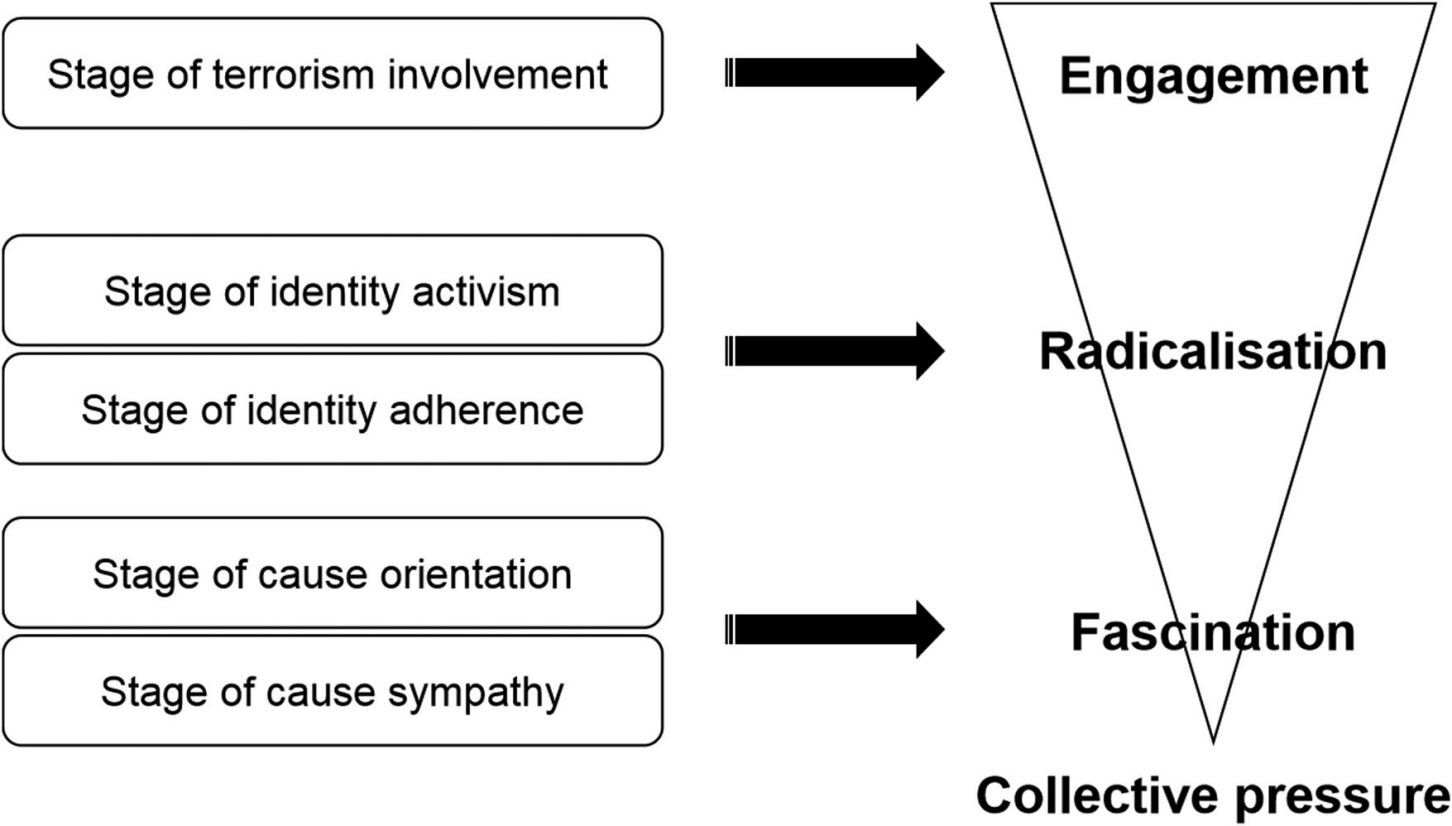
Model of cognitive and affective self-definition and meaning construction in violent radical engagement.

Subsequently, we reconciled these descriptions of the psychological functioning of the persons involved in the radicalisation process with existing models, and defined three phases: fascination, radicalisation and terrorist involvement (Figure 1).

The fascination phase comprises the first two radicalisation stages, which translate the dynamic of reconciliation through the subjective analysis of the specific attributes of the radical cause and the positive valence that the person gives it according to his/her own history. At this stage, the person usually adopts the position of a victim, characterised by a negative individualism fed by the biased representations of persecution, misunderstanding, injustice and demands.

The radicalisation phase also comprises two parts. In the first, the person evolves toward identity adherence, which is characterised by proactively taking steps to look for peers while distancing him/herself from traditional referents (e.g. family, school, other associations). The person also adopts an increasingly polarised view of the world. The emergence of activism follows the process of engagement and identity affirmation. Activism can be described as peri-democratic to the extent that it is situated on the fringes of social and democratic norms. This level of identity adherence is principally the sounding board that constitutes a group of peers at this stage of the radicalisation process. The person is no longer only reinforced by their own representations in relation to themselves, or by the effect they believe they have on their environment, but also by the positive feedback they receive from external observers, whose adherence to the cause emphasises and reinforces the choices made. This collective pressure from the radical group, represented in Figure 1 by an inverted triangle that describes the increasing influence, rests on a dialectic, non-coercive process in which social modelling and stronger interactions progressively align the interpretive context proposed by the group and the interpretive system of the person. At this stage of identity adherence, the subject moves beyond the phase of fascination, in which the radical topic remained external, even if the subject tended to bring it closer to merge with it and assimilate it into his/her definition of identity.

The engagement phase is defined at the sociocognitive level by a loss of inhibitory mechanisms and a difficulty in backtracking. This is because questioning the choices made would create cognitive dissonance that is difficult for an individual to manage, given the lack of differentiation between personal and collective goals.

## Discussion

Our modelling of the cognitive and affective self-definition and meaning construction in violent radical engagement addresses the phenomenon of radicalisation from a psychological perspective and clarifies the cognitive processes underlying the motives of young individuals who have left their country to join wars to further the Islamic State or to commit terrorist attacks. At present, even if the Califate were defeated, numerous terrorist attacks would still be committed. However, the operating mode of such attacks has changed. The dissipation of ISIS has deprived terrorist attacks planned in Western countries of the logistics necessary to realise large-scale violent acts. As a result, the recent terrorist attacks seem mostly expressions of radicalised individuals who are less involved in complex networks of recruitment. The instability and fragility of the personality structures that seem to characterise recent profiles of perpetrators of terrorist acts, together with an apparent lack (or minimum) of links with structured networks, indicate that the collective pressure of radical groups, which was noticeable during earlier radicalisation and engagement phases, is now weaker. To understand these changes and the reasons individual commit such attacks, it is necessary to examine the frustration mechanisms related to the fascination phase (i.e. the initial phase of the radicalisation process).

The fascination phase reflects a passage from a “simple” feeling of a shared radical Islamic cause to a proactive step toward it. The main radicalisation models assume that this step begins with a personal (affective component) and collective frustration, which results from a subjective comparison of perceived disparities and inequalities (cognitive component) [45].

This comparison process generates a self-perception “in the negative” [46], which tends to change into a victim attitude based on the person’s perceived lack of something that “he/she has not” or “he/she is not”. This differentiation is associated with a negative charge. The negative identity associated with this victim attitude is nevertheless a source of positive reinforcement and gratification, as it contributes to a social identity and feeling of belonging to a certain group, even if that group is perceived as discriminated against. This attitude legitimises the radical statements of the group with which the person tries to create proximity, which is mostly imagined at this stage. In fact, external reasons for persecution, incomprehension and injustice (which combine to generate a perception of discrimination on behalf of the exogenous group) prevent the person experiencing cognitive discrepancy. The justification of existential suffering caused by such external factors transforms the feeling of frustration resulting from negative comparisons, feelings and emotions (e.g. anger or frustration) into an expression of a right and legitimate revolt, sometimes even redemption. The accumulation of positive attributions related to the evaluative component stimulates the cognitive component of identity adherence through the person’s recognition of his/her adherence to the cause. At this stage, the affective component is often composed of individual considerations and emotional aspirations that also reinforce the identification.

Even more than the radicalisation and engagement phases, this first phase of fascination for the radical cause establishes a breach that expresses a radical interpretation through reformulation of the moral basis induced by the transformation of the victim attitude. In fact, this attitude legitimises the claim and the use of non-democratic and violent means, even if at this stage the violence is perpetrated by others and the person adhering to the cause is still a spectator. The reformulation of the moral basis that becomes possible owing to numerous cognitive mechanisms of neutralisation and moral disengagement (which are present from this first phase) trivialises the use of violence, which becomes a necessary and legitimate method to affirm one’s social identity in a public space (and is considered a lawful method, as it is a consequence of the perceived injustice inflicted). The proximity to radicalisation induced by this restructuring of representations facilitates and strengthens subsequent active information seeking or, in the absence of network links, promotes activism or eventual action.

In most recent terrorist attacks, the perpetrators’ personal psychological problems, a source of frustration that is not necessarily pathological, supplemented the radical affirmation of legitimate violence that is characteristic of the victim attitude and through the mechanism of narcissistic self-reinforcement rapidly created the intention to commit an attack. In these cases, the radical statement of the action, which was more than ideological, can be seen as a pretext and a meaningful justification of an egocentric and deadly action that reflected personal identity problems.

Despite the prospects it opens up, this study has several limitations. The first limitation, which is specific to this field of research, concerns the definition of the concept of radicalisation. It is very difficult to define the nature and the boundaries of this process. For this study, we chose to conceptualise the radicalisation process as part of a continuum. However, this approach is contestable, specifically in relation to Islamic radicalisation for ideological and political reasons. The second limitation concerns the data source. We used data from legal expert interviews. This approach raises questions about the compliance and sincerity of the subjects, given the legal issues that surrounded them. Finally, we adopted an inductive clinical and qualitative approach based on individual interviews that is regularly used in criminology. Although this approach provides rich information, and permitted investigation of the sociocognitive mechanisms underlying the radicalisation process, it ideally requires quantitative confirmation to ensure the validity of the model identified.

## Conclusion

Although psychological models of radicalisation have been previously discredited, current approaches within the sociocognitive paradigm of personality offer a conceptual framework that can increase understanding of how a person’s cognitive (as well as environmental) information analysis can affect the radicalisation process.

This qualitative and inductive research referred to central studies in the field of radicalisation and sought to elucidate the cognitive aspect of radicalisation. The descriptive characteristics of the subjects were entirely in accord with previous findings. Using a qualitative approach, we were able to develop a model that allows the investigation of the sociocognitive mechanisms involved in the interpretive systems of radicalised people. This model of cognitive and affective transformation of self-definition and sense construction in violent radical engagement considers the process of radicalisation from the individual’s point of view through successive modifications induced by a gradual cognitive restructuring in a context of dynamic interactions and mutual determinism with the environment. This model also allows the integration of the transformation of subjective representations related to feelings of injustice and discrimination. These feelings are mobilised by the person’s interpretative system when they assess their own situation and form the basis for motivation toward radicalism. This level of analysis seems essential to us, as it allows us to understand how different levels of macro- and meso-analysis, whatever their content, are mobilised and integrated into the person’s thought system. We are aware of the study limitations: the data were based on expert assessments of individuals involved in the process of radicalisation and who were often involved in the judicial and/or penal systems; the use of such data raises several concerns, including that of compliance. However, access to this population of radicalised people remains very difficult, and few studies use such interviews, even if these are within a framework of forensic expertise. However, we believe that these data are valuable as, to our knowledge, this is the only study on the sociocognitive processes underlying radicalisation. We aim to conduct further, quantitative, studies to test this model to ensure that it has empirical validity and a strong conceptual basis.
